# Genomic Analysis of an Indian G8P[1] Caprine Rotavirus-A Strain Revealing Artiodactyl and DS-1-Like Human Multispecies Reassortment

**DOI:** 10.3389/fvets.2020.606661

**Published:** 2021-01-27

**Authors:** Shubhankar Sircar, Yashpal Singh Malik, Prashant Kumar, Mohd Ikram Ansari, Sudipta Bhat, S. Shanmuganathan, Jobin Jose Kattoor, O.R. Vinodhkumar, Narayan Rishi, Nadia Touil, Souvik Ghosh, Krisztián Bányai, Kuldeep Dhama

**Affiliations:** ^1^Division of Biological Standardization, Indian Council of Agricultural Research-Indian Veterinary Research Institute, Bareilly, India; ^2^Amity Institute of Virology and Immunology, J-3 Block, Amity University, Noida, India; ^3^College of Animal Biotechnology, Guru Angad Dev Veterinary and Animal Sciences University, Ludhiana, India; ^4^Animal Disease Diagnsotic Laboratory, Purdue University, West Lafayette, IN, United States; ^5^Division of Epidemiology, Indian Council of Agricultural Research-Indian Veterinary Research Institute, Bareilly, India; ^6^Laboratoire de Recherche et de Biosécurité, Hôpital Militaire d'instruction Med V de Rabat, Rabat, Morocco; ^7^Department of Biomedical Sciences, One Health Center for Zoonoses and Tropical Veterinary Medicine, Ross University School of Veterinary Medicine, Basseterre, Saint Kitts and Nevis; ^8^Centre for Agricultural Research, Institute for Veterinary Medical Research, Hungarian Academy of Sciences, Budapest, Hungary; ^9^Division of Pathology, Indian Council of Agricultural Research-Indian Veterinary Research Institute, Bareilly, India

**Keywords:** rotavirus-A, goat, G8P[1] strain, whole-genome analysis, India, reassortment

## Abstract

The surveillance studies for the presence of caprine rotavirus A (RVA) are limited in India, and the data for the whole-genome analysis of the caprine RVA is not available. This study describes the whole-genome-based analysis of a caprine rotavirus A strain, RVA/Goat-wt/IND/K-98/2015, from a goat kid in India. The genomic analysis revealed that the caprine RVA strain K-98, possess artiodactyl-like and DS-1 human-like genome constellation G8P[1]-I2-R2-C2-M2-A3-N2-T6-E2-H3. The three structural genes (VP2, VP4, and VP7) were close to caprine host having nucleotide-based identity range between 97.5 and 98.9%. Apart from them, other gene segments showed similarity with either bovine or human like genes, ultimately pointing toward a common evolutionary origin having an artiodactyl-type backbone of strain K-98. Phylogenetically, the various genes of the current study isolate also clustered inside clades comprising Human-Bovine-Caprine isolates from worldwide. The current findings add to the knowledge on caprine rotaviruses and might play a substantial role in designing future vaccines or different alternative strategies combating such infections having public health significance. To the best of our knowledge, this is the first report on the whole-genome characterization of a caprine RVA G8P[1] strain from India. Concerning the complex nature of the K-98 genome, whole-genome analyses of more numbers of RVA strains from different parts of the country are needed to comprehend the genomic nature and genetic diversity among caprine RVA.

## Introduction

Rotaviruses (RVs) are the major viral pathogens that often leads to severe diarrhea in young neonates of animals as well as humans. It affects multiple livestock species, including newborn calves (1), pigs (2), foals (3), small ruminants (4), incurring significant economic losses (5). The virus is a non-enveloped, triple-layered, and the genome consists of 11 segmented dsRNA encoding for six structural genes and six non-structural proteins (6). Based on antigenic and genetic properties, RVs are further classified into eight recognized species *A-H* and two tentative species rotavirus *I* and rotavirus *J* (7, 8).

As per recommendations of the Rotavirus Classification Work Group (RCWG), nucleotide percent similarity cut-off values of all 11 viral gene segments are used to determine a genotypic scheme Gx–P[x]–Ix–Rx–Cx–Mx–Ax–Nx–Tx–Ex–Hx designating VP7–VP4–VP6–VP1–VP2–VP3–NSP1–NSP2–NSP3–NSP4–NSP5/6 genes, respectively (9, 10).

Globally, data regarding epidemiology, prevalence, and circulating genotypes of small ruminant RVA is scarce compared to other domesticated species. To date, three rotavirus species (RVA, RVB, and RVC) have been reported in the caprine population worldwide (11, 12). Association of RVs with diarrhea in the caprine population and their prevalence have been described from the United Kingdom (13), Italy (14), Spain (15), Japan (16), South Korea (16), Egypt (17), Bangladesh (18), Turkey (19), and Argentina (20). Apart from these published reports, a few GenBank sequence records of caprine RVA are available from Argentina (21), China (22), and India (23) but are unpublished. To best of our knowledge, there are only two reports from India describing the detection and characterization of caprine RVA (24, 25). Past studies reported G2, G3, G6, G8, G9, and G10 and with P[1], P[3], P[4], P[5], P[8], P[11], P[14] and P[15] genotype circulation in caprine and ovine population worldwide (26). To sum up the count of whole-genome reports available for caprine RVA, there are only four complete genomes published from Argentina (20), Bangladesh (18), China (22), and Uganda (27).

Based on the 20th livestock census, the combined cattle and buffalo population of India stands at 302.34 million (56.42%), and small ruminants constitute around 223.14 million (41.64%) in livestock, which are a notable species reared by the marginal farmers for their livelihood. Goats contribute around 27.8% (148.88 million) of livestock. India lacks epidemiological and molecular studies on caprine RVs. The proximity of the caprine population to human settlements is a matter of concern with regards to the interspecies transmission of caprine and human RVA between them (27, 28). Several studies have documented zoonotic as well as zooanthroponotic transmission of common RV genotypes between the caprine-bovine-human population (18, 29–32). We, for the first time characterized the whole-genome of a caprine isolate and studied its possible origin. The findings of this study contribute to the understanding of re-assortment events, interspecies transmission, and the emergence of unusual RVA isolates.

## Materials and Methods

### Sampling, Screening, and Primers Designing

During an investigation of the outbreak of diarrhea in goat population located in an unorganized sector, reared by local people randomly, a sample K-98 was collected from a goat kid in the Northern state of Uttar Pradesh, India was found positive using an antigen-capture sandwich ELISA for RVA infection (33).

In the preliminary testing, RNA–polyacrylamide gel electrophoresis (34, 35) revealed eleven dsRNA bands of RVA with 4:2:3:2 pattern after silver staining.

The sample was processed by preparing 10% (w/v) fecal suspension in 1X PBS, followed by RNA extraction using Qiazol lysis reagent (Qiagen, Hilden, Germany). The extracted RNA was then reverse-transcribed using MuMLV RT (Promega, Wisconsin, USA). PCR for the detection/diagnosis of RVA was carried out using previously described primers based on the VP6 gene, the positive samples yielded a specific amplicon of 226 bp on agarose gel electrophoresis (36). Structural genes VP2, VP3, VP4, VP6, VP7, and non-structural genes NSP1, NSP2, NSP3, NSP4, and NSP5 were amplified using earlier reported primers (18, 37, 38) whereas two new pair of overlapping primers for VP1 gene were designed to amplify its partial gene for nearly ~1,400 bp (Supplementary Data 1). Standalone software like MEGA 7.0 (39) and online primer designing tool IDTs Oligoanalyzer (https://eu.idtdna.com/calc/analyzer) were used to design and verify the primers sets (33).

### Complete Genome Amplification—Cloning, Sequencing, and Genotyping

The PCR amplified products of all the gene segments were cloned in pDrive vector (Qiagen, Hilden, Germany) and sequenced bi-directionally with universal sequencing primer pairs T7 and SP6. The open reading frames (ORFs) along with respective untranslated regions (UTR) were located using the EditSeq tool, DNASTAR software (Lasergene, USA) for all the gene segments. The VP7 gene is responsible for eliciting neutralizing antibodies through its antigenic epitopes. The VP7 trimer consists of two structurally distinct antigenic epitopes: 7-1 and 7-2. The 7-1 epitope extends to the inter-subunit boundary and is further subdivided into 7-1a and 7-1b (40). Comparison between the amino acid residues that contains the 7-1a, 7-1b, and 7-2 epitopes of the strain K-98 and other G8 type VP7 genes from different species was conducted using the MegAlign tool of DNASTAR (**Figure 4**). All eleven gene segments were assigned a particular genotype by comparing them against the available reference sequences in NCBI GenBank using the nucleotide BLAST tool (BLASTn) search. Further, their genotype was also ascertained using the online Rotavirus A Genotype Determination tool (https://www.viprbrc.org). Nearest isolates to eleven gene segments were also determined and compared using the BLAST tool and RVA genotyping tool of ViPRBRC (https://www.viprbrc.org).

### NCBI GenBank Submissions

Each gene segment for the caprine strain RVA/Goat-wt/IND/K-98/2015 was submitted to NCBI GenBank under the accession number MT501452 to MT501462.

### Phylogenetic Analysis and Percent Identity Calculation

Phylogenetic analysis was conducted using MEGA 7.0 software (39) using the maximum likelihood (ML) method. Before applying the ML method, best substitution models were selected according to the individual datasets of all the 11 gene segment through “find model” program incorporated in MEGA 7.0. Based on the “find model” analysis, datasets of six genes (NSP1, NSP2, VP1, VP2, VP3, and VP7) were assigned the GTR+G+I substitution model, whereas four genes (NSP3, NSP4, VP3, and VP4) and one single gene (NSP5) were assigned GTR+G and HKY+G substitution models, respectively.

### Recombination Detection

*In silico* study was performed to identify the possible chances of recombination in the virus genes using the recombination detection program 4 (RDP 4 v 4.95) (41). Following the detection of a “recombination signal” with BOOTSCAN (42), MAXCHI (43), CHIMERA (44), 3SEQ (45), GENECONV (46), LARD (47), and SISCAN (48), RDP4 determines approximate breakpoint positions using a Hidden Markov Model, BURT. It then identifies the recombinant sequence using the PHYLPRO (49), VISRD (50), and EEEP methods (51, 52). The RDP 4 program was used for all the 11 gene segments to find out any possible recombination.

### Selection Pressure Analysis

The datamonkey web server (http://www.datamonkey.org/), was used to analyze the major genes (VP7, VP4, VP6, and NSP4) for selection pressure analysis to test the rate of non-synonymous to synonymous (dN/dS) ratio (53). Several codon-specific models like Fixed-Effect-Likelihood (FEL), Single Likelihood Ancestor Counting (SLAC), Mixed Effects Model of Evolution (MEME), and Fast, Unconstrained Bayesian AppRoximation were used for Inferring Selection (FUBAR) methods incorporated in the datamonkey web server. Sites identified significant by at least three models were considered under positive selection.

## Results

### Whole-Genome Sequencing and Genotype Constellation

The 11 genes of the RVA including six structural genes VP1, VP2, VP3, VP4, VP6, VP7, and five non-structural genes NSP1, NSP2, NSP3, NSP4, and NSP5 for the strain K-98 were sequenced and compared with other RVA sequences available in the GenBank database. Nucleotide sequencing analysis in ViPR and BLASTn analysis of each gene revealed a genotype constellation G8P[1]-I2-R2-C2-M2-A13-N2-T6-E2-H3. The lengths of the nucleotide sequences obtained for each of the 11 genomic segments of strain K-98 are shown in Table 1.

**Table 1:** Size and accession number of 11 gene segments of caprine strain RVA/Goat-wt/IND/K-98/2015.

**Sl No**.	**Gene name**	**Size (bp)**	**Accession no**.	**Nucleotide completeness**	**ORFs position**
1	VP1	1,402	MT501452	Partial cds	12–1,402
2	VP2	2,663	MT501453	Complete cds	17–2,662
3	VP3	2,586	MT501454	Complete cds	9–2,516
4	VP4	2,087	MT501455	Partial cds	1–2,087
5	VP6	1,314	MT501456	Complete cds	9–1,202
6	VP7	981	MT501457	Complete cds	1–981
7	NSP1	1,515	MT501458	Complete cds	3–1,478
8	NSP2	1,060	MT501459	Complete cds	47–1,000
9	NSP3	1,074	MT501460	Complete cds	26–967
10	NSP4	739	MT501461	Complete cds	42–569
11	NSP5/NSP6	654	MT501462	Complete cds	14–610/72–368

**Figure 1:**
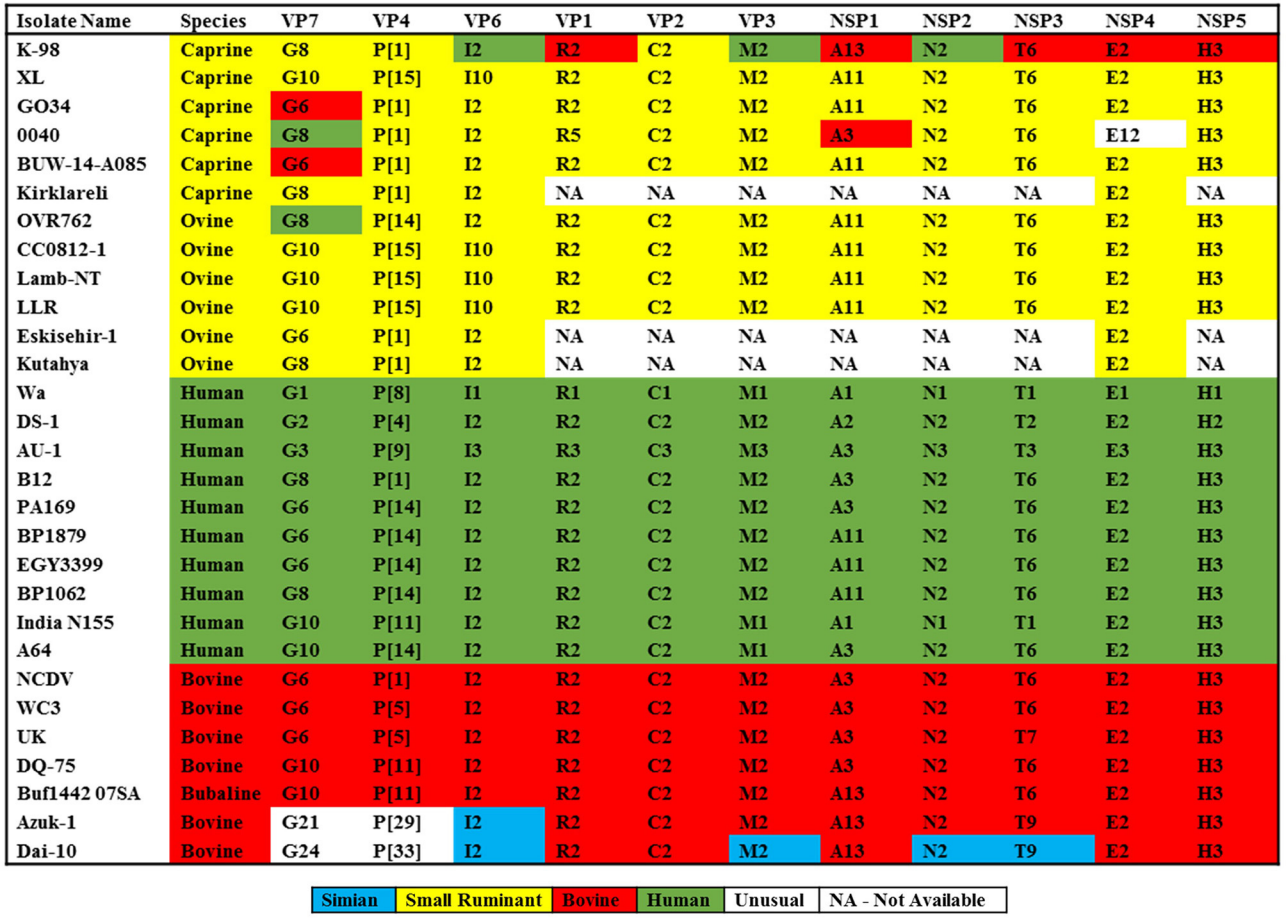
Comparison of genomic constellations of RVA/Goat-wt/IND/K-98/2015 with those of selected human and animal RVA strains with known genomic constellations. Individual gene segments of all strains are color coded based on the maximum homology with the RVA strains available in the public domain.

All genotypes have been reported earlier, and its comparison to known genotype constellations of human, bovine, and small ruminant are shown in Figure 1. The genotype constellation of strain K-98 reveal a mixed backbone of RVA wherein five genes (VP1, NSP1, NSP3, NSP4, and NSP5) were derived from a bovine host, three genes (VP3, VP6, and NSP2) were derived from a human host, and three caprine gene segments (VP2, VP4, and VP7) were derived from caprine species (Figure 1). Based on the genotype constellation, it was observed that apart from NSP1, where an unusual bovine like A13 genotype was observed, all other genes exhibited genotypes that have been described in the caprine host in earlier studies. The genomic combination of G8P[1] has also been reported in the Argentinian caprine population earlier (20). Reports of G8P[1] genotype being diagnosed in small ruminants of Turkey emerged in 2012 and recently in 2020 (19, 54).

### Phylogenetic and Percent Identity Analysis of Non-structural Proteins (NSP1-5)

**Figure 2:**
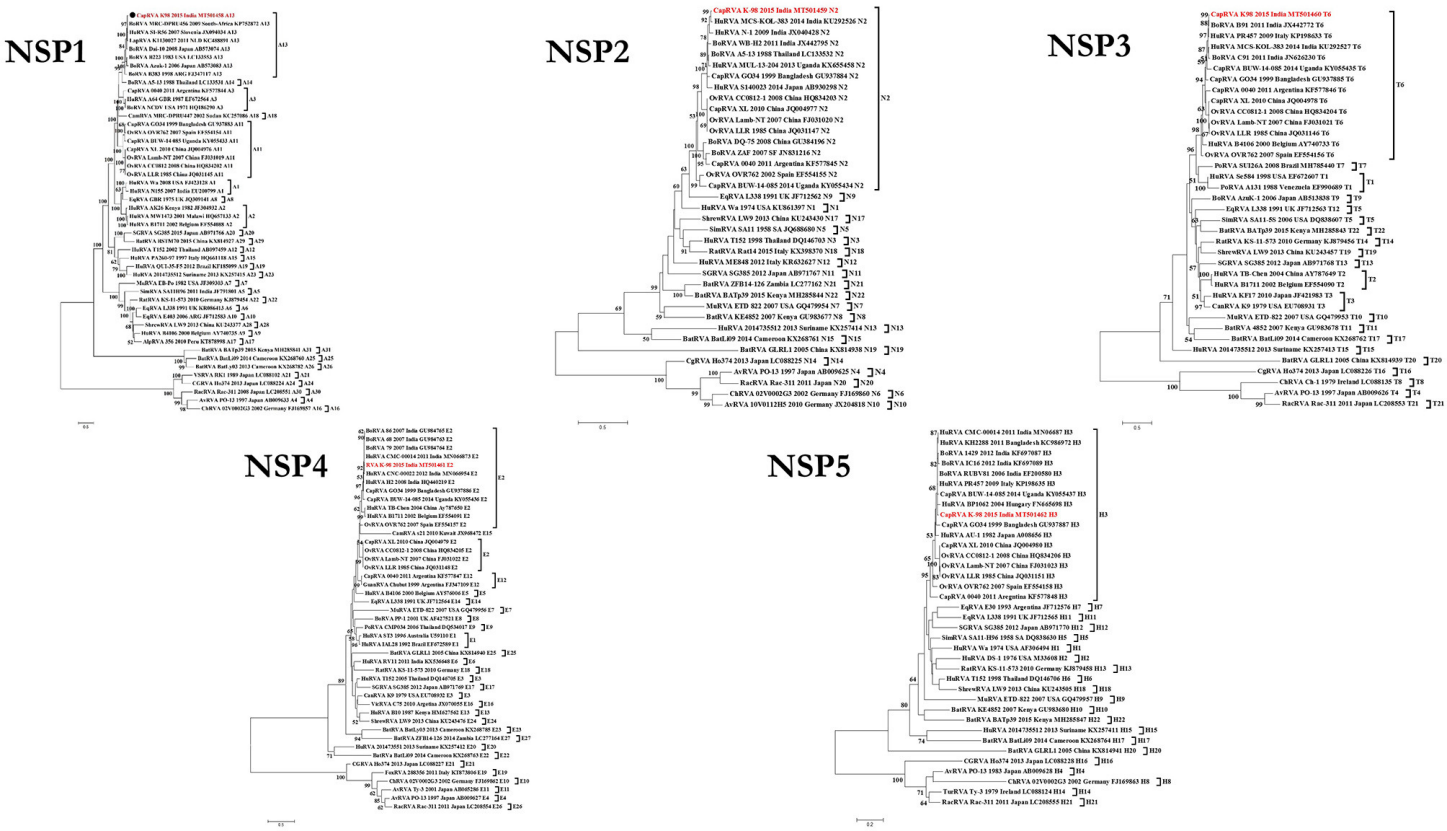
Phylogenetic dendrograms of all non-structural gene segments of caprine strain RVA/Goat-wt/IND/K-98/2015/G8P[1] with those of representative RVA strains. Phylogenetic dendrogram was constructed by the maximum likelihood method using MEGA software version 7. Phylogenetic distances were statistically supported by bootstrapping with 1,000 replicates. In all the dendrograms, the position of strain K-98 is indicated by the red colored text. The scale bar indicates nucleotide substitutions per site. The genotypes are indicated at the right-hand side of each dendrogram.

In phylogenetic analysis, K-98 NSP1 gene clustered with A13 type bovine and bovine-like human (SI-R56) and Lapine (K1130027) RVA isolates (Figure 2). Rest all other small ruminant isolates from worldwide clustered inside a single clade of A11 genotype. The nucleotide sequence of the NSP1 gene for K-98 showed the maximum similarity of 95.7% with a bovine isolate from South Africa (KP752872) (Supplementary Data 2). Strain K-98 showed 94.2% nucleotide similarity with its next nearest neighbor, a bovine-like Lapine, and human RVA isolates.

In the phylogenetic analysis of the NSP2 gene, the isolate K-98 formed a separate cluster along with human RVA isolates MCS-KOL-383 and N-1 from India inside the N2 genotype clade (Figure 2). All major small ruminant strains from worldwide also clustered inside the same branch of the N2 genotype. It showed the highest percent nucleotide similarity of 98.1% with a human isolate (KU292526) from Kolkata, India. Further, the percent nucleotide similarity with the nearest human isolate was 94.7% (KX655458) from Uganda, whereas it was found to be 94.6% (JX040428) with a caprine isolate from Bangladesh, respectively (Supplementary Data 2).

The NSP3 gene of isolate K-98 clustered inside a clade comprising other small ruminant isolates belonging to the T6 genotype (Figure 2). The NSP3 gene of K-98 isolate showed 99.5% identity with a bovine isolate BR91 (JX442772) from India, which is an artiodactyl-like human RVA strain. Apart from BR91, the next nearest neighbor to the K-98 NSP3 gene was an Italian human isolate PR457 (KP198633), showing 98.6% similarity (Supplementary Data 2).

The NSP4 gene of K-98 isolate also grouped inside a clade comprising other E2 genotype isolates from bovine, human, and caprine species (Figure 2). Apart from E2 type NSP4, E12 genotype has been previously described in Argentinian caprine RVA isolate 0040. Notably, a camel RVA (JX968472) carrying E15 NSP4 genotype appeared inside the major clade of E2. The NSP4 gene of K-98 was closely related to a bovine isolate 86 (GU984765) of western India, sharing a nucleotide percent identity of 98.4% (Supplementary Data 2).

In the phylogenetic analysis of the NSP5 gene segment, K-98 clustered alongside humans, bovine, caprine, and ovine RVA isolates inside the major clade of the H3 genotype. It showed the highest percent nucleotide similarity of 99.0% with bovine isolate RUBV81 (EF200580) from India (Supplementary Data 2). The nearest caprine isolate was Ugandan BUW-14-085 (KY055437), with a nucleotide percent identity of 98.4% (Figure 2).

### Phylogenetic and Percent Identity Analysis of Structural Proteins (VP1-4 and VP6-7)

**Figure 3:**
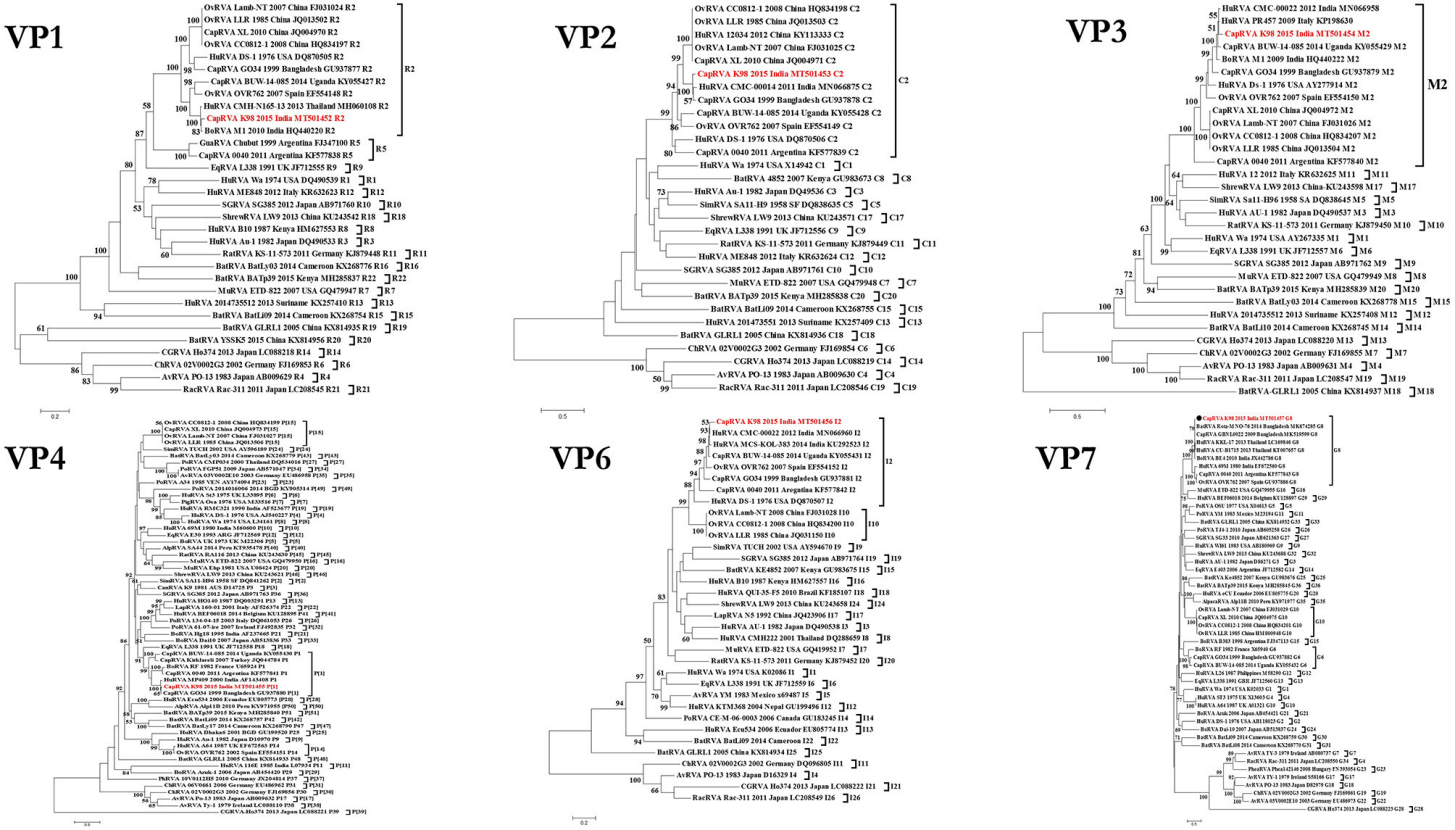
Phylogenetic dendrograms of all structural gene segment of caprine strain RVA/Goat-wt/IND/K-98/2015/G8P(1) with those of representative RVA strains. Phylogenetic dendrogram was constructed by the maximum likelihood method using MEGA software version 7. Phylogenetic distances were statistically supported by bootstrapping with 1,000 replicates. In all the dendrograms, the position of strain K-98 is indicated by the red-colored text. The scale bar indicates nucleotide substitutions per site. The genotypes are indicated at the right-hand side of each dendrogram.

Phylogenetic analysis of the VP1 gene segment revealed that all the small ruminant RVA were found to fall in the R2 genotype clade except the Argentinian caprine strain 0040 (KF577838), which possessed the R5 genotype. The current study isolate K-98 clustered with an Indian bovine rotvairus A BoRVA isolate M1 (HQ440220), which also shared the highest similarity of 97.5% at the nucleotide level. The percent similarity with human, ovine, and caprine isolates ranged from 88.5 to 88.9%, belonging to group R2 genotype (Figure 3 and Supplementary Data 2).

The VP2 gene of isolate K-98 is grouped within the C2 genotype clade with all other small ruminant strains worldwide (Figure 3). Caprine strain GO34 (GU937878) from Bangladesh was the nearest neighbor in the phylogeny having 97.5% nucleotide similarity (Supplementary Data 2). Apart from GO34, an Indian human RVA isolate having a 95.3% identity at the nucleotide level also appeared inside the same branch (Figure 3). The percent nucleotide similarity with other isolates like the prototype DS-1 human RVA isolate was 95.3%, whereas it ranged between 85.5 and 89.4% with small ruminant origin strains worldwide.

The phylogenetic analysis of the VP3 gene clustered the K-98 isolate close to two human isolates from Italy and India inside a major clade comprising the M2 genotype (Figure 3). Apart from PR457 human RVA isolate from Italy, it clustered alongside an Indian human RVA isolate CMC-00022 (MN066958) with a nucleotide similarity of 96.9%. The M2 genotype clade is also comprised of small ruminant origin isolates from worldwide sharing close relationships. The percent nucleotide identity of the VP3 gene of the K-98 isolate showed the highest similarity of 97.5% with a human RVA isolate PR457 (KP198630) from Italy (Supplementary Data 2).

In a phylogenetic analysis of the outer capsid VP6 gene, K-98 clustered alongside human RVA isolates from eastern and southern India within the I2 clade (Figure 3). The I2 clade also includes the caprine and ovine origin strains from China, Bangladesh, Uganda, Spain, and Argentina. The percent nucleotide similarity of K-98 showed the highest similarity of 97.4% with a human RVA isolate MCS-KOL-383 (KU292523) from India (Supplementary Data 2).

The phylogenetic analysis of the VP4 gene grouped the caprine RVA K-98 isolate with caprine and a human RVA isolate of P[1] genotype having the highest similarity of 97.4% at the nucleotide level with a caprine RVA isolate GO34 (GU937878) from Bangladesh (Supplementary Data 2 and Figure 3). Apart from our study, P[1] genotype have been reported from Bangladesh, Argentina, and Ugandan caprine strains. Genotype P[1] has been the major P-type reported worldwide in small ruminant, whereas P[14] and P[15] has only been reported in Spain and China, respectively.

**Figure 4:**
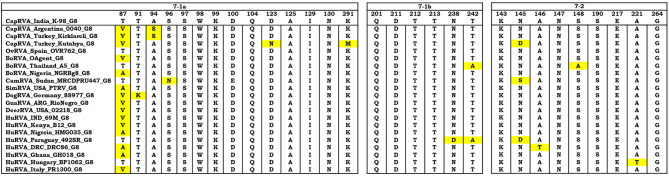
The VP7 antigenic epitopes analysis of caprine strain RVA/Goat-wt/IND/K-98/2015 with selected G8 strains. The alignment of residues in VP7 is divided into three antigenic epitopes (7-1a, 7-1b, and 7-2). Residues that differ from the strain K-98 caprine strain are highlighted by yellow color. Majority of the residues were observed to be in consensus with the caprine strain K-98 except the site 87 where extensive variation was observed throughout the species.

In the phylogenetic analysis, VP7 gene of the K-98 isolate clustered into a clade comprising G8 genotype strains (Figure 3). Apart from the current study, small ruminant origin G8 genotype strains have been reported from Argentinian caprine strain 0040, Spanish, and Turkish ovine strain OVR762 and Kutahya, respectively. Other G-types of small ruminants that clustered separately in the phylogenetic tree include G6 and G10. The VP7 gene of the K-98 isolate shows the highest percent nucleotide identity with bat isolate Rota_MNO_76 (MK674285) from Bangladesh. The next nearest neighbor to isolate K-98 is a Bangladeshi caprine RVA isolate GBNL0022. Moreover, the maximum nucleotide identity with other G8 type RVA species like caprine, human, bovine, and the ovine isolate was 98.9, 98.6, 98.1, and 85.5%, respectively (Supplementary Data 2). The major antigenic epitopes of the VP7 gene were also analyzed with those of other reported G8 genotypes of different species worldwide (Figure 4). Among all the 29 antigenic epitopes, residues number 87 showed extensive change concerning other G8 genotype reported in different species.

### Recombination Detection by RDP 4.0

**Figure 5:**
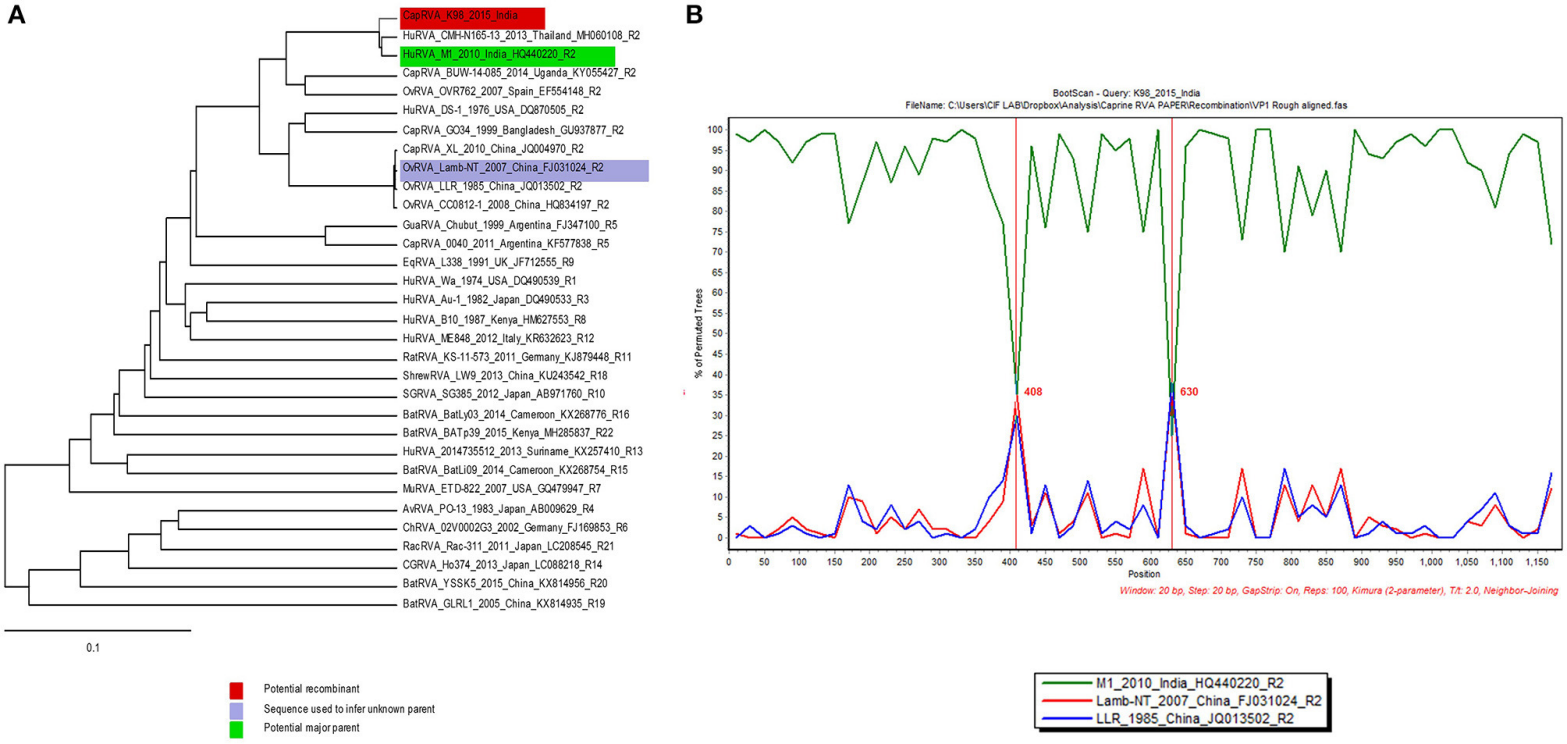
SimPlot output for VP1 gene segment of caprine RVA strain K-98. **(A)** The image shows RDP 4.0 output depicting the major and minor parents by blue and green color on a phylogenetic tree. **(B)** The right-side window in the image generated using SimPlot analysis shows the recombination crossover points (408 and 630) in the gene depicted by a green and blue color line across them.

Analysis of all the genes from K-98 isolate for any possible recombination revealed recombination events in segment 1 (VP1) gene. The statistical results supported that K-98 (isolated in this study), HuRVA_M1_2010 (GenBank no. HQ440220), and OvRVA_Lamb-NT_2007 (GenBank no. FJ031024) are three recombinants exhibiting unique genetic recombination patterns (recombinant score >0.68, *P* < 0.001) (Supplementary Data 1). Recombination events were further confirmed by SimPlot analysis (55). SimPlot analysis detected recombination breakpoints at position number 408 and 630 (Figure 5).

### Selection Pressure Analysis

Using the datamonkey web server, site by site-selective pressure analysis by FEL, SLAC, MEME, and FUBAR methods were tried with no positively selected sites in any of the four genes chosen for the analysis. For each of the four genes, either one or two models gave positive selection sites, but as per the thumb rule, those sites which are identified as significant by at least three models are only considered positively selected. In the current study, none of the genes showed a positive selection at least by three models.

## Discussion

RVs are the major diarrheic pathogens which causes severe diarrhea in humans as well as domesticated animals. There have been ample reports regarding RVA infection in large ruminants from different parts of the world, but data concerning RVA infections in small ruminants (sheep and goats) is very scarce. We aimed to investigate RVA associated diarrhea in the goat population of India. In India, only few studies have been done on RVA infection in goat population (24, 25). Apart from these two previous studies, there is only an NCBI GenBank submission for a partial length VP7 gene segment (KC416965) of the G8 genotype from India in the year 2011. Following a shortage in reports of RVA infection status in goat population of India, the data on its characterization is also less. The scarcity of well-characterized caprine RVA strains in NCBI GenBank from goat species India also needs to be addressed. This report characterizes the first whole-genome of a caprine isolate RVA/Goat-wt/IND/K-98/2015 from India.

Upon characterization of the caprine RVA isolate K-98, it possessed the genotype constellation of G8P[1]-I2-R2-C2-M2-A13-N2-T6-E2-H3, which is exhibited by RVA strains human and artiodactyl (bovine, caprine, and ovine) type species (9, 28, 56). Human G8P[1], G10P[11], G6P[14], G8P[14], and G10P[14] strains also display this type of consensus genotype constellation (29, 57–60). Previously, whole-genome caprine RVA strains reported from China, Bangladesh, Argentina and Uganda showed multi-reassortant backbone wherein few genes were also found to have been derived from human. Majority, of the previously described caprine RVA backbones, were showing closeness to artiodactyl-type species where they were not significantly associated with any other rotavirus strains known till date.

In the phylogenetic analysis of non-structural protein gene segments of K98, NSP1, NSP3, NSP4, and NSP5 were found clustering with bovine isolates, whereas only NSP2 is branching alongside a human RVA isolate. Gene segment NSP1 and NSP5 of K-98 clustered with bovine RVA isolates from South Africa (MRC-DPRU456) and India (RUBV81) having 95.7% and 99.0% of nucleotide similarity, respectively. It was observed that these two bovine RVA isolates from South Africa (61) and India (62) are only NCBI GenBank records for which no publications were found. Therefore, their origin and association with a particular species could not be determined. Gene segment NSP2 was located closer to Indian human RVA isolate KOL-383 (KU292526) having a close identity with Indian human rotavirus A HuRVA strain N-1 and Bangladeshi caprine RVA strain GO34 possessing human origin (63). Thus, NSP2 reflects a true human-origin having 98.2% nucleotide similarity with HuRVA isolate KOL-383 (KU292526). Gene segment NSP3 was found closer to a bovine RVA isolate HR-B91 in phylogenetic analysis having an artiodactyl-like backbone with 99.5% of nucleotide similarity (32). Similarly, gene segment NSP4 shared a high nucleotide similarity of 98.4% with a bovine RVA isolate 86 (GU984765) clustering alongside three human-like bovine RVA isolates from western India (64).

In the structural protein gene analysis, the VP1 gene of the current study isolate K-98 clustered with an Indian BoRVA isolate M1 (HQ440220) (65) within the R2 clade sharing the highest similarity of 97.5% at the nucleotide level. This particular bovine RVA isolate M1 has been described to be close to Italian human strains. The VP2 gene of isolate K-98 grouped with caprine GO34 (GU937878) and human CMC-00014 isolates from Bangladesh (18) and India (66) inside the C2 genotype clade having 97.5 and 95.3% nucleotide similarity, respectively. The study regarding the caprine isolate GO34 indicated its closeness to a Chinese lamb isolate Lamb-NT (67), which suggests it to be of small ruminant origin. Similarly, the VP4 gene was also found closer to caprine GO34 isolate in the phylogenetic analysis, which also shared a high nucleotide-based identity of 97.4%. The GO34 VP4 gene sequence was found closer to a bovine isolate A5 from Thailand. Two gene segments VP3 and VP6 also clustered close to human RVA isolates in phylogeny having 97.5% and 97.4% nucleotide-based identity. Moreover, the human RVA isolate PR457 close to VP3 K-98 had been described to possess a bovine-like VP3 gene in its respective publication. Similarly, the human RVA isolate KOL-383 from Kolkata, India also contained a porcine/bovine like VP6 gene. In a phylogenetic analysis of the VP7 gene of the K-98 isolate clustered into a clade comprising G8 genotype strains. It showed the highest percent nucleotide identity with bat isolate Rota_MNO_76 (MK674285) from Bangladesh (68). This caprine RVA VP7 G8 isolate from Bangladesh was described to have transmitted from human or livestock to bats. It shared high a high similarity to human, bovine as well as porcine RVA strains. As reported in the recently concluded study, the closeness of VP7 gene of K-98 to this bat RVA isolate from Bangladesh points toward the possibility that bats may have acquired the RVA infection as they share the water bodies while drinking which lie in close proximity to livestock as well as humans (68). The next nearest neighbor to isolate K-98 is a Bangladeshi caprine RVA isolate GBNL0022, which also appeared to be a GenBank submission (69).

Phylogenetic and percent identity analysis indicated that five gene segments (NSP1, NSP3, NSP4, NSP5, and VP1) and three gene segments (NSP2, VP3, and VP6) out of the eleven gene segments of caprine strain K-98 were derived from a heterologous host species, bovine and human, respectively. Notably, the RNA binding protein VP2 and the two surface protein genes VP4 and VP7, which are responsible for stimulating the production of neutralizing antibodies, were found to be of caprine origin. Cumulatively, it was observed that out of the eleven gene segments, 4 NSPs (NSP1, NSP3, NSP4, NSP5) and 6 VPs (VP1-VP7) were found having their origin from artiodactyl-like RVA strains.

In the antigenic epitope analysis of VP7, apart from the residue number 87, all other residues in the three putative antigenic regions showed that the VP7 gene of strain K-98 might have shared origin with human, bovine, and small ruminant origin G8 genotype. There were very few sporadic changes in the antigenic epitope regions, which point toward the conservation of pivotal epitopes that are responsible for attachment of the virus to the host.

Upon analyzing the two major neutralizing genes VP4 and VP7, along with the background data, we observed that G8P[1] is quite a common genotype circulating in caprine and ovine population, which is also found in humans, cattle, monkey, guanaco, goats, dogs, and other hosts (19, 54, 60, 70, 71). In Asia, Central America and Europe, this G8P[1] genotype combination has also been described to possess bovine-like gene segments in different mammalian species (72–75). In India, a partial G8 genotype has also been reported from North India in the year 2011, which had been submitted to NCBI GenBank (ca/KRR81/IVRI). The G8 genotype has been published worldwide in artiodactyl type species (ruminants and camelids) (20, 71). It was also described earlier in Hungary, where a zoonotic RVA strain had spread from goat and sheep to human (76). Although G8 genotype is quite commonly found in humans (77), the close proximity of humans and goats to each other may be responsible for the host switching of G8 genotype. Moreover, the finding of genotype A13 of NSP1, which is usually a common bovine genotype also sheds lights toward the interspecies transmission of artiodactyl-type genes among different farm animals.

Recombination analysis by RDP and Simplot software revealed putative recombination events in gene segment VP1. It was observed to have recombined with an Indian human-like bovine and a Chinese lamb RVA strains. These *in-silico* based analyses suggest the continuous recombination tendency of RVA strains having zoonosis potential between human and caprine origin RVA strains.

## Conclusion

The first full-length genome sequencing of a K-98 caprine isolate from India revealed a complex bovine or a human reassortant RVA strain backbone. In total, the strain K-98 possessed an artiodactyl type genomic constellation, which seems to have acquired the majority of genes from bovine, porcine, or caprine host. Due to these conjectures, it is tough to determine the particular RVA backbone's exact ancestral origin. Diarrheal diseases due to rotavirus have been a significant burden to the large ruminant industry. Still, the burden of such etiologies in the small ruminant industry has not been explored widely in previous studies from India.

Due to the simultaneous circulation of identical RVA genotypes in humans, large and small ruminants, the utmost importance for the surveillance studies targeting these three species is needed in the future. The full genomic analysis of strain K-98 has provided significant insights into the whole genetic makeup of caprine RVA strain from India and its genetic relatedness to different RVA from other host species. Concerning the complex nature of the K-98 genome, whole-genome analyses of RVA strains from different parts of the country are needed to comprehend the genomic nature and genetic diversity of caprine RVA. The current study's findings add to the knowledge on caprine rotaviruses and might play a substantial role in designing future vaccines or other alternative strategies combating such infections having public health significance.

## Data Availability Statement

The datasets generated in this study can be found in online repositories. The names of the repository/repositories and accession number(s) can be found in the article/Supplementary Material.

## Ethics Statement

The study involves non-invasive methods of sample collection therefore no ethical issues were involved.

## Author Contributions

SSi and YM involved in the conceptualization of work. SSi, MA, SB, and OV involved in the analysis of data and manuscript drafting. SSh and JJK helped in sampling and drafting of initial manuscript. PK performed the computational studies, and did manuscript drafting. NT helped in reviewing the manuscript, SG and KB helped in writing and reviewing of the manuscript. YM and KD worked on final draft check and finalization of submission. All authors contributed to the article and approved the submitted version.

## Conflict of Interest

The authors declare that the research was conducted in the absence of any commercial or financial relationships that could be construed as a potential conflict of interest.

## References

[1] Saif L Jiang B. Nongroup A rotaviruses of humans and animals. Curr Top Microbiol Immunol. (1994) 185:339–71. 10.1007/978-3-642-78256-5_11 8050284

[2] Malik Y Kumar N Sharma K Sircar S Dhama K Bora DP Rotavirus diarrhea in piglets: a review on epidemiology, genetic diversity and zoonotic risks. Indian J Anim Sci. (2014) 84:1035–42.

[3] Conner ME Darlington R. Rotavirus infection in foals. Am J Vet Res. (1980) 41:1699–703. 6261616

[4] Martella V Decaro N Buonavoglia C Enteric viral infections in lambs or kids. Vet Microbiol. (2015) 181:154–60. 10.1016/j.vetmic.2015.08.006 26321129 PMC7131559

[5] Dhama K Chauhan R Mahendran M Malik S. Rotavirus diarrhea in bovines and other domestic animals. Vet Res Commun. (2009) 33:1–23. 10.1007/s11259-008-9070-x 18622713 PMC7088678

[6] Estes M Kapikian A Fields Virology. Philadelphia, PA: Lippencott, Williams and Wilkins (2007).

[7] Bányai K Kemenesi G Budinski I Földes F Zana B Marton S. Candidate new rotavirus species in Schreiber's bats, Serbia. Infect Genet Evol. (2017) 48:19–26. 10.1016/j.meegid.2016.12.002 27932285 PMC7106153

[8] Mihalov-Kovács E Gellért Á Marton S Farkas SL Fehér E Oldal M. Candidate new rotavirus species in sheltered dogs, Hungary. Emerg Infect Dis. (2015) 21:660. 10.3201/eid2104.141370 25811414 PMC4378476

[9] Matthijnssens J Ciarlet M Rahman M Attoui H Bányai K Estes MK. Recommendations for the classification of group A rotaviruses using all 11 genomic RNA segments. Arch Virol. (2008) 153:1621–9. 10.1007/s00705-008-0155-1 18604469 PMC2556306

[10] RCWG Rotavirus Classification Working Group: Newly Assigned Genotypes: RCWG. (2019). Available online at: https://rega.kuleuven.be/cev/viralmetagenomics/virus-classification/rcwg (accessed September 27, 2020).

[11] Munoz M Alvarez M Lanza I Carmenes P. Role of enteric pathogens in the aetiology of neonatal diarrhoea in lambs and goat kids in Spain. Epidemiol Infect. (1996) 117:203–11. 10.1017/S0950268800001321 8760970 PMC2271684

[12] Kaminjolo J Adesiyun A. Rotavirus infection in calves, piglets, lambs and goat kids in Trinidad. Br Vet J. (1994) 150:293–9. 10.1016/S0007-1935(05)80009-0 8044669 PMC7130288

[13] Scott A Luddington J Lucas M Gilbert F. Rotavirus in goats. Vet Rec. (1978) 103:145. 10.1136/vr.103.7.145-b 211701

[14] Legrottaglie R Volpe A Rizzi V Agrimi P. Isolation and identification of rotaviruses as aetiological agents of neonatal diarrhoea in kids. Electrophoretical characterization by PAGE. New Microbiol. (1993) 16:227–35. 8396195

[15] Munoz M Alvarez M Lanza I Carmenes P. An outbreak of diarrhoea associated with atypical rotaviruses in goat kids. Res Vet Sci. (1995) 59:180–2. 10.1016/0034-5288(95)90057-8 8525112

[16] Lee J-B Youn S-J Nakagomi T Park S-Y Kim T-J Song C-S. Isolation, serologic and molecular characterization of the first G3 caprine rotavirus. Arch Virol. (2003) 148:643–57. 10.1007/s00705-002-0963-7 12664291

[17] Khafagi M Mahmoud M Habashi A Prevalence of rotavirus infections in small ruminants. Global Vet. (2010) 4:539–43.

[18] Ghosh S Alam MM Ahmed MU Talukdar RI Paul SK Kobayashi N. Complete genome constellation of a caprine group A rotavirus strain reveals common evolution with ruminant and human rotavirus strains. J Gen Virol. (2010) 91:2367–73. 10.1099/vir.0.022244-0 20505013

[19] Alkan F Gulyaz V Timurkan MO Iyisan S Ozdemir S Turan N. A large outbreak of enteritis in goat flocks in Marmara, Turkey, by G8P [1] group A rotaviruses. Arch Virol. (2012) 157:1183–7. 10.1007/s00705-012-1263-5 22367501

[20] Uriarte ELL Badaracco A Matthijnssens J Zeller M Heylen E Manazza J The first caprine rotavirus detected in Argentina displays genomic features resembling virus strains infecting members of the Bovidae and Camelidae. Vet Microbiol. (2014) 171:189–97. 10.1016/j.vetmic.2014.03.013 24742949

[21] de Beer M Steele D Characterization of the VP7 and VP4 Genes of a South African Group A Caprine Rotavirus (GenBank Record). Bethesda, MD: NCBI (2002).

[22] Liu F Xie JX Liu CG Wang KG Zhou BJ Wen M Full Genomic Sequence and Phylogenetic Analyses of a Caprine G10P[15] Rotavirus A Strain XL Detected in 2010 (GenBank Record). Huaxi, GZ: NCBI (2010).

[23] Kaur S Bhilegaonkar KN Dubal ZB Rawat S Lokesh KM Caprine Rotavirus A Isolate Ca/KRR81/IVRI/India/2011/G8 Outer Capsid Protein (VP7) Gene, Partial cds (GenBank Record). Bethesda, MD: NCBI (2013).

[24] Reddy G Kumari A Mishra A Shivashanarappa N Paul S Gupta V Prevalence of groupa rotavirus in diarrhoeic goat kids from organized goat farms. Indian J Comp Immunol Microbiol Infect Dis. (2014) 35:9–12.

[25] Singh U Singh R Singh AP Yadav SK Sircar S Malik YS Detection and characterization of caprine and ovine rotaviruses, India. Indian J Anim Sci. (2017) 87:1358–61.

[26] Papp H Malik YS Farkas SL Jakab F Martella V Bányai K. Rotavirus strains in neglected animal species including lambs, goats and camelids. Virusdisease. (2014) 25:215–22. 10.1007/s13337-014-0203-2 25674588 PMC4188177

[27] Bwogi J Jere KC Karamagi C Byarugaba DK Namuwulya P Baliraine FN. Whole genome analysis of selected human and animal rotaviruses identified in Uganda from 2012 to 2014 reveals complex genome reassortment events between human, bovine, caprine and porcine strains. PLoS ONE. (2017) 12:e0178855. 10.1371/journal.pone.0178855 28640820 PMC5480867

[28] Matthijnssens J Potgieter CA Ciarlet M Parreño V Martella V Bányai K Are human P [14] rotavirus strains the result of interspecies transmissions from sheep or other ungulates that belong to the mammalian order Artiodactyla? J Virol. (2009) 83:2917–29. 10.1128/JVI.02246-08 19153225 PMC2655590

[29] Ghosh S Varghese V Samajdar S Sinha M Naik TN Kobayashi N. Evidence for bovine origin of VP4 and VP7 genes of human group A rotavirus G6P [14] and G10P [14] strains. J Clin Microbiol. (2007) 45:2751–3. 10.1128/JCM.00230-07 17537935 PMC1951218

[30] Rajendran P Kang G. Molecular epidemiology of rotavirus in children and animals and characterization of an unusual G10P [15] strain associated with bovine diarrhea in south India. Vaccine. (2014) 32:A89–94. 10.1016/j.vaccine.2014.03.026 25091687

[31] Doro R Farkas SL Martella V Bányai K. Zoonotic transmission of rotavirus: surveillance and control. Expert Rev Anti Infect Ther. (2015) 13:1337–50. 10.1586/14787210.2015.1089171 26428261

[32] Kumar N Malik Y Sharma K Dhama K Ghosh S Bányai K. Molecular characterization of unusual bovine rotavirus A strains having high genetic relatedness with human rotavirus: evidence for zooanthroponotic transmission. Zoonoses Public Health. (2018) 65431–42. 10.1111/zph.12452 29464925

[33] Kumar N Malik YS Kumar S Sharma K Sircar S Saurabh S. Peptide-recombinant VP6 protein based enzyme immunoassay for the detection of group a rotaviruses in multiple host species. PLoS ONE. (2016) 11:e0159027. 10.1371/journal.pone.0159027 27391106 PMC4938596

[34] Laemmli UK. Cleavage of structural proteins during the assembly of the head of bacteriophage T4. Nature. (1970) 227:680–5. 10.1038/227680a0 5432063

[35] Malik YS Sharma K Vaid N Chakravarti S Chandrashekar K Basera SS. Frequency of group A rotavirus with mixed G and P genotypes in bovines: predominance of G3 genotype and its emergence in combination with G8/G10 types. J Vet Sci. (2012) 13:271–8. 10.4142/jvs.2012.13.3.271 23006956 PMC3467402

[36] Mondal A Sharma K Malik YS Joardar SN Detection of group a rotavirus in faeces of diarrhoeic bovine porcine and human population from eastern India by reverse transcriptase-polymerase chain reaction. Adv Anim Vet Sci. (2013) 1:18–9.

[37] Gentsch JR Glass R Woods P Gouvea V Gorziglia M Flores J. Identification of group A rotavirus gene 4 types by polymerase chain reaction. J Clin Microbiol. (1992) 30:1365–73. 10.1128/JCM.30.6.1365-1373.1992 1320625 PMC265294

[38] Ghosh S Kobayashi N Nagashima S Chawla-Sarkar M Krishnan T Ganesh B. Full genomic analysis and possible origin of a porcine G12 rotavirus strain RU172. Virus Genes. (2010) 40:382–8. 10.1007/s11262-010-0454-y 20157771

[39] Kumar S Stecher G Tamura K. MEGA7: molecular evolutionary genetics analysis version 7.0 for bigger datasets. Mol Biol Evol. (2016) 33:1870–4. 10.1093/molbev/msw054 27004904 PMC8210823

[40] Aoki ST Settembre EC Trask SD Greenberg HB Harrison SC Dormitzer PR. Structure of rotavirus outer-layer protein VP7 bound with a neutralizing Fab. Science. (2009) 324:1444–7. 10.1126/science.1170481 19520960 PMC2995306

[41] Martin DP Murrell B Golden M Khoosal A Muhire B. RDP4: detection and analysis of recombination patterns in virus genomes. Virus Evol. (2015) 1:vev003. 10.1093/ve/vev003 27774277 PMC5014473

[42] Salminen MO Carr JK Burke DS McCutchan FE. Identification of breakpoints in intergenotypic recombinants of HIV type 1 by bootscanning. AIDS Res Hum Retroviruses. (1995) 11:1423–5. 10.1089/aid.1995.11.1423 8573403

[43] Smith JM. Analyzing the mosaic structure of genes. J Mol Evol. (1992) 34:126–9. 10.1007/BF00182389 1556748

[44] Posada D Crandall KA. Evaluation of methods for detecting recombination from DNA sequences: computer simulations. Proc Natl Acad Sci USA. (2001) 98:13757–62. 10.1073/pnas.241370698 11717435 PMC61114

[45] Boni MF Posada D Feldman MW. An exact nonparametric method for inferring mosaic structure in sequence triplets. Genetics. (2007) 176:1035–47. 10.1534/genetics.106.068874 17409078 PMC1894573

[46] Padidam M Sawyer S Fauquet CM. Possible emergence of new geminiviruses by frequent recombination. Virology. (1999) 265:218–25. 10.1006/viro.1999.0056 10600594

[47] Holmes EC Worobey M Rambaut A. Phylogenetic evidence for recombination in dengue virus. Mol Biol Evol. (1999) 16:405–9. 10.1093/oxfordjournals.molbev.a026121 10331266

[48] Gibbs MJ Armstrong JS Gibbs AJ. Sister-scanning: a Monte Carlo procedure for assessing signals in recombinant sequences. Bioinformatics. (2000) 16:573–82. 10.1093/bioinformatics/16.7.573 11038328

[49] Weiller GF. Phylogenetic profiles: a graphical method for detecting genetic recombinations in homologous sequences. Mol Biol Evol. (1998) 15:326–35. 10.1093/oxfordjournals.molbev.a025929 9501499

[50] Lemey P Lott M Martin DP Moulton V. Identifying recombinants in human and primate immunodeficiency virus sequence alignments using quartet scanning. BMC Bioinformatics. (2009) 10:126. 10.1186/1471-2105-10-126 19397803 PMC2684544

[51] Beiko RG Hamilton N. Phylogenetic identification of lateral genetic transfer events. BMC Evol Biol. (2006) 6:15. 10.1186/1471-2148-6-15 16472400 PMC1431587

[52] Heath L Van Der Walt E Varsani A Martin DP. Recombination patterns in aphthoviruses mirror those found in other picornaviruses. J Virol. (2006) 80:11827–32. 10.1128/JVI.01100-06 16971423 PMC1642601

[53] Weaver S Shank SD Spielman SJ Li M Muse SV Kosakovsky Pond SL. Datamonkey 2.0: a modern web application for characterizing selective and other evolutionary processes. Mol Biol Evol. (2018) 35:773–7. 10.1093/molbev/msx335 29301006 PMC5850112

[54] Timurkan MÖ Alkan F. Identification of rotavirus A strains in small ruminants: first detection of G8P [1] genotypes in sheep in Turkey. Arch Virol. (2020) 165:425–31. 10.1007/s00705-019-04476-7 31828508

[55] Lole KS Bollinger RC Paranjape RS Gadkari D Kulkarni SS Novak NG. Full-length human immunodeficiency virus type 1 genomes from subtype C-infected seroconverters in India, with evidence of intersubtype recombination. J Virol. (1999) 73:152–60. 10.1128/JVI.73.1.152-160.1999 9847317 PMC103818

[56] Matthijnssens J Ciarlet M Heiman E Arijs I Delbeke T McDonald SM. Full genome-based classification of rotaviruses reveals a common origin between human Wa-Like and porcine rotavirus strains and human DS-1-like and bovine rotavirus strains. J Virol. (2008) 82:3204–19. 10.1128/JVI.02257-07 18216098 PMC2268446

[57] Ramani S Iturriza-Gomara M Jana AK Kuruvilla KA Gray JJ Brown DW. Whole genome characterization of reassortant G10P [11] strain (N155) from a neonate with symptomatic rotavirus infection: identification of genes of human and animal rotavirus origin. J Clin Virol. (2009) 45:237–44. 10.1016/j.jcv.2009.05.003 19505846 PMC2913240

[58] Bányai K Papp H Dandár E Molnár P Mihály I Van Ranst M. Whole genome sequencing and phylogenetic analysis of a zoonotic human G8P [14] rotavirus strain. Infect Genet Evol. (2010) 10:1140–4. 10.1016/j.meegid.2010.05.001 20471499

[59] El Sherif M Esona MD Wang Y Gentsch JR Jiang B Glass RI. Detection of the first G6P [14] human rotavirus strain from a child with diarrhea in Egypt. Infect Genet Evol. (2011) 11:1436–42. 10.1016/j.meegid.2011.05.012 21640199

[60] Ghosh S Gatheru Z Nyangao J Adachi N Urushibara N Kobayashi N. Full genomic analysis of a G8P [1] rotavirus strain isolated from an asymptomatic infant in Kenya provides evidence for an artiodactyl-to-human interspecies transmission event. J Med Virol. (2011) 83:367–76. 10.1002/jmv.21974 21181935

[61] Das SR Halpin RA Stucker KM Akopov A Fedorova N Puri V Rotavirus A Strain RVA/Cow-wt/ZAF/MRC-DPRU456/2009/G6P[11] Segment 5 Non-Structural Protein 1 (NSP1) Gene—KP752872 (Genbank Record). Bethesda, MD: NCBI (2015).

[62] Ghosh S Naik TN NSP5-Encoding Gene of a Bovine Group A Rotavirus Strain—EF200580 (GenBank Record). Bethesda, MD: NCBI (2006).

[63] Mandal P Mullick S Nayak MK Mukherjee A Ganguly N Niyogi P. Complete genotyping of unusual species A rotavirus G12P [11] and G10P [14] isolates and evidence of frequent *in vivo* reassortment among the rotaviruses detected in children with diarrhea in Kolkata, India, during 2014. Arch Virol. (2016) 161:2773–85. 10.1007/s00705-016-2969-6 27447463

[64] Chitambar SD Arora R Kolpe AB Yadav MM Raut CG. Molecular characterization of unusual bovine group A rotavirus G8P[14] strains identified in western India: emergence of P[14] genotype. Vet Microbiol. (2011) 148:384–8. 10.1016/j.vetmic.2010.08.027 20880637

[65] Malik YS Kumar N Sharma K Saurabh S Dhama K Prasad M. Multispecies reassortant bovine rotavirus strain carries a novel simian G3-like VP7 genotype. Infect Genet Evol. (2016) 41:63–72. 10.1016/j.meegid.2016.03.023 27033751

[66] Tan G Pickett B Fedorova N Amedeo P Hu L Christensen J Rotavirus A Strain RVA/Human-wt/IND/CMC_00014/2011/G6P[X] Segment 2 Core Capsid Protein VP2 (VP2) Gene, Complete cds—MN066875 (Genbank Record). Bethesda, MD: NCBI (2011).

[67] Chen Y Zhu W Sui S Yin Y Hu S Zhang X. Whole genome sequencing of lamb rotavirus and comparative analysis with other mammalian rotaviruses. Virus Genes. (2009) 38:302–10. 10.1007/s11262-009-0332-7 19214729

[68] Islam A Hossain ME Rostal MK Ferdous J Islam A Hasan R. Epidemiology and molecular characterization of rotavirus A in fruit bats in Bangladesh. EcoHealth. (2020) 17:398–405. 10.1007/s10393-020-01488-7 32876756 PMC7464061

[69] Hossain MB Rahman MS Hasan R Hossain ME Rahman MZ Rahman M Rotavirus in ruminants of Bangladesh: epidemiology and molecular characterization—MK519599 (GenBank Record). Bethesda, MD: NCBI (2009).

[70] Jere KC Mlera L O'Neill HG Peenze I van Dijk AA. Whole genome sequence analyses of three African bovine rotaviruses reveal that they emerged through multiple reassortment events between rotaviruses from different mammalian species. Vet Microbiol. (2012) 159:245–50. 10.1016/j.vetmic.2012.03.040 22541163

[71] Komoto S Adah MI Ide T Yoshikawa T Taniguchi K. Whole genomic analysis of human and bovine G8P [1] rotavirus strains isolated in Nigeria provides evidence for direct bovine-to-human interspecies transmission. Infect Genet Evol. (2016) 43:424–33. 10.1016/j.meegid.2016.06.023 27302094

[72] Jagannath M Vethanayagam RR Reddy BY Raman S Rao CD. Characterization of human symptomatic rotavirus isolates MP409 and MP480 having 'long'RNA electropherotype and subgroup I specificity, highly related to the P6 [1], G8 type bovine rotavirus A5, from Mysore, India. Arch Virol. (2000) 145:1339–57. 10.1007/s007050070094 10963341

[73] Martinez M Phan TG Galeano ME Russomando G Parreno V Delwart E. Genomic characterization of a rotavirus G8P [1] detected in a child with diarrhea reveal direct animal-to-human transmission. Infect Genet Evol. 2014;27:402-7. 10.1016/j.meegid.2014.08.015 25169054

[74] Sieg M Rückner A Köhler C Burgener I Vahlenkamp TW. A bovine G8P [1] group A rotavirus isolated from an asymptomatically infected dog. J Gen Virol. (2015) 96:106–14. 10.1099/vir.0.069120-0 25304653

[75] Taniguchi K Urasawa T Pongsuwanna Y Choonthanom M Jayavasu C Urasawa S. Molecular and antigenic analyses of serotypes 8 and 10 of bovine rotaviruses in Thailand. J Gen Virol. (1991) 72:2929–37. 10.1099/0022-1317-72-12-2929 1662688

[76] Marton S Dóró R Fehér E Forró B Ihász K Varga-Kugler R. Whole genome sequencing of a rare rotavirus from archived stool sample demonstrates independent zoonotic origin of human G8P [14] strains in Hungary. Virus Res. (2017) 227:96–103. 10.1016/j.virusres.2016.09.012 27671785

[77] Agbemabiese CA Nakagomi T Doan YH Nakagomi O. Whole genomic constellation of the first human G8 rotavirus strain detected in Japan. Infect Genet Evol. (2015) 35:184–93. 10.1016/j.meegid.2015.07.033 26275468

